# Association of Lymphopenia with Short Term Outcomes of Sepsis Patients; a Brief Report

**Published:** 2019-01-20

**Authors:** Hojat Sheikh Motahar Vahedi, Aida Bagheri, Amirhosein Jahanshir, Javad Seyedhosseini, Elnaz Vahidi

**Affiliations:** 1 Prehospital Research Center, Tehran University of Medical Sciences, Tehran, Iran.; 2 Department of Emergency Medicine, Shariati Hospital, Tehran University of Medical Sciences, Tehran, Iran.

**Keywords:** Lymphopenia, sepsis, prognosis, emergency service, hospital

## Abstract

**Introduction:**

Studies have claimed that low lymphocyte count is independently correlated with 28-day survival of sepsis patients. Therefore, this study aimed to evaluate the value of lymphopenia in predicting the short-term outcome of sepsis patients.

**Methods:**

This cross-sectional study was performed on sepsis patients referred to the emergency department during an 8-month period and relationship of lymphopenia with 28-day mortality and probability of septic shock and readmission due to sepsis was assessed.

**Results:**

124 cases with the mean age of 66.12 15.82 (21-90) years were studied (54.8% male). 81 (65.3%) cases had lymphopenia (59.3% male). Lymphopenic patients had a significantly higher mean age (p = 0.003), higher need for ICU admission (p < 0.001), higher prevalence of 28-day septic shock (p < 0.001), higher 28-day mortality (p < 0.001), higher probability of readmission due to sepsis (p = 0.048), and higher SOFA score (p < 0.001). During 28 days of follow up, 57 (46%) patients were expired. They had a higher prevalence of septic shock (p < 0.001) and higher SOFA score (p < 0.001). Multivariate analysis showed that septic shock (OR=364.6; 95% CI: 26.3 to 5051.7; p = 0.001) and lymphopenia (OR=19.2; 95% CI: 1.7 to 211.3; p = 0.016) were the independent predictors of 28-day mortality.

**Conclusions:**

Based on the findings, lymphopenia was independently associated with higher 28-day mortality and lymphopenic patients were older than the control group and had a significantly higher need for ICU admission, higher probability of 28-day septic shock and readmission due to sepsis, and higher SOFA score.

## Introduction:

Sepsis is one of the most common causes of emergency department (ED) referral among all different ages, especially elderlies. It is reported that 571000 severe sepsis cases are annually referred to EDs in the US. Its mortality risk is between 20% and 50% and it is among the 10^th^ most common reasons of mortality and morbidity in US (1-3). 

Currently, it is believed that sepsis triggers a complicated immunologic response that impairs the balance between pro and anti-inflammatory processes. This results in an immune suppression phase leading to progressive primary and secondary infections, and high morbidity and mortality rates (7). One of the immune suppression characteristics in sepsis is apoptosis of immune cells including T-helper and cytotoxic lymphocytes, B lymphocytes and dendritic cells (8). Many studies have shown that lymphocyte count decreases in the early phase of sepsis and follows the same pattern during the first 28 days (8-13). 

It was reported that neutrophilia and lymphopenia were both related to bacteremia, but the latter had a higher predictive value. It is also found that both neutrophilia and lymphopenia had more prognostic value than total white blood cell (WBC) count (14). It was also claimed that low lymphocyte count independently correlates with 28-day patient survival rate in sepsis and some special types of organ failure (15). Based on the above-mentioned points, this study aimed to evaluate the value of lymphopenia in predicting the short-term outcome of sepsis patients.

## Methods:

***Study Design and setting***

This cross-sectional study was performed on sepsis patients referred to emergency department of Shariati Hospital, Tehran, Iran, during the first 8 months of 2017. The study method was approved by the ethics committee of Tehran University of Medical Sciences and the code of IR.TUMS.MEDICINE.REC.1395.1627 has been assigned. Signed informed written consent has been obtained from all patients or their family to participate in our study. 

***Participants***

Sampling was performed prospectively in a continuous manner and all patients over 18 years old suspected of having sepsis who were admitted to the ED were eligible. Concomitant underlying diseases affecting white blood cell (WBC) count like blood dysplasia, history of immune deficiency or being under chemotherapy, death before the 4^th^ day, and unwillingness to participate in our research were considered as exclusion criteria. 

***Data gathering***

A predesigned checklist including the demographic information of patients as well as source of infection, sequential organ failure assessment (SOFA) score, length of hospital stay, need for intensive care unit (ICU) admission, lymphocyte count, and 28-day outcome was filled for all patients by a senior emergency medicine resident.

Peripheral blood samples were taken on the admission day and 4 days later, and complete blood count (CBC) and cell differentiation were performed. Lymphopenia was defined as lymphocyte count <1500 cells/L and lymphocyte count ≥1500 cells/L was considered normal. This count should have remained in the same group during the first 4 days of admission, and the cases whose count changed over 4 days were excluded. 

28-day mortality, 28-day readmission due to sepsis, and 28-day prevalence of septic shock were considered as outcomes.

***Statistical analysis***

SPSS v.22 was used for statistical analyses. Descriptive statistics, including mean ± standard deviation or frequency (percentage) were used for reporting the findings. Comparison of baseline characteristics between 28-day survivors and expired patients was done using unpaired t-test, Mann-Whitney U test, or chi-square test, as appropriate. To determine the independent effect of absolute lymphocyte count on 28-day mortality, logistic regression analysis was used. P < 0.05 was considered as level of significance.

## Results:

143 patients suspected of having sepsis were studied, 124 of whom met the inclusion criteria. The mean age of the participants was 66.12 15.82 (21-90) years (54.8% male). 81 (65.3%) cases had lymphopenia (59.3% male). Table 1 compares the characteristics of lymphopenic patients with others. Lymphopenic patients had a significantly higher mean age (p = 0.003), higher need for ICU admission (p < 0.001), higher prevalence of 28-day septic shock (p < 0.001), higher 28-day mortality (p < 0.001), higher probability of readmission due to sepsis (p = 0.048), and higher SOFA score (p < 0.001). During 28 days of follow up, 57 (46%) patients were expired. Table 2 compares the characteristics of dead and survived patients. The mean age of the expired patients was significantly higher (p=0.001). They had a significantly higher rate of lymphopenia (p < 0.001), higher prevalence of septic shock (p < 0.001), and higher SOFA score (p < 0.001). Multivariate analysis showed that septic shock (OR=364.6; 95% CI: 26.3 to 5051.7; p = 0.001) and lymphopenia (OR=19.2; 95% CI: 1.7 to 211.3; p = 0.016) were independent significant predictors of 28-day mortality. 

## Discussion

Based on the findings, lymphopenia was independently associated with higher 28-day mortality and lymphopenic patients were older than the control group and had a significantly higher need for ICU admission, higher probability of 28-day septic shock and readmission due to sepsis, and higher SOFA score. 

**Table 1:** Comparing the characteristics of lymphopenic sepsis patients with non-lymphopenic ones

**Variables**	**Lymphocyte count**		**P value**
	**< 1500 (n = 81)**	**≥ 1500 (n = 43)**	
**Sex (%)**			
Male	48 (59.3)	20 (46.5)	0.182
Female	33 (40.7)	22 (51.2)	
**Age (year)**			
18 - 45	4 (4.9)	8 (18.6)	0.042
45 - 60	16 (19.8)	9 (20.9)	
≥ 60	61 (75.3)	26 (60.5)	
**Duration of admission (day)**			
Mean ± SD	10.90 ± 6.67	9.79 ± 5.81	0.359
**Need for ICU admission**			
Yes	42 (51.9)	6 (14.0)	< 0.001
No	39 (48.1)	37 (86.0)	
**Source of infection**			
Pneumonia	50 (61.7)	23 (53.5)	0.914
Urinary tract	19 (23.5)	12 (27.9)	
Soft tissue	4 (4.9)	3 (17.0)	
Central nervous system	1 (1.2)	1 (2.3)	
Others	7 (8.6)	4 (9.3)	
**SOFA score**			
Mean ± SD	5.74 ± 2.58	3.90 ± 2.22	< 0.001
**28-day outcome**			
Mortality	59 (88.1)	8 (11.9)	< 0.001
Septic shock	57 (85.1)	10 (14.9)	< 0.001
Readmission	20 (52.6)	18 (47.4)	0.048

Data are presented as mean ± standard deviation (SD) or number (%).

**Table 2:** Comparing the characteristics of expired and survived groups

**Variables**	**Died (n=67)**	**Survived (n=57)**	**P-value**
**Sex**			
Male	37 (55.4)	31 (54.4)	0.553
Female	30 (44.8)	26 (45.6)	
**Age (year)**			
Mean ± SD	73.10 ± 12.41	57.91 ± 15.54	0.001
**Source of infection (%)**			
Pneumonia	44 (65.7)	29 (50.9)	0.652
Urinary tract	14 (20.9)	17 (29.9)	
Soft tissue	3 (4.5)	4 (7.1)	
Central nervous system	1 (1.5)	1 (1.5)	
Others	5 (7.4)	6 (10.6)	
**Septic shock **			
Number (%)	64 (95.5)	3 (5.3)	0.001
**Lymphocyte count**			
Mean ± SD	991.7 684.7	1923.8 1167.9	0.001
Median (IQR)	847.0 (784.0)	1600.0 (1011.0)	
Lymphopenia<1500 (%)	59 (88.1)	22 (38.6)	
**SOFA score**			
Mean ± SD	6.9 2.0	2.9 0.9	0.001
Median (IQR)	6.0 (4.0)	3.0 (1.0)	

IQR: Interquartile range; SD: standard deviation; SOFA: sepsis related organ failure assessment. Data are presented as mean ± standard deviation (SD) or number (%).

Some previous studies like those of Cheadle et al., Felmet et al., Monserrat et al., Hein et al., and Inoue et al. confirmed that B and T cell lymphocyte counts had significantly reduced during the first week of diagnosis in patients who died of sepsis (8-12).

In 2014, Drewry et al. studied 335 adult patients with bacteremia and sepsis and reported the death of 77 cases within 28 days after admission. They showed that the median lymphocyte count on the 4^th^ day of admission was a good independent predictor in mortality prediction (4). 

In 2015, Chung et al. announced that severe lymphopenia was associated with elevated plasma interleukin (IL)-15 levels and increased mortality during severe sepsis (1). They considered severe lymphopenia as lymphocyte count less than 0.510^3 ^cells/l and found that it was associated with increased 28-day mortality in severe sepsis. Like in our study, pneumonia and urinary tract infections were the most common sources of sepsis in the population they studied. They reported that septic shock had a higher incidence rate in the expired group. Expired group had a higher SOFA score compared to survivors.

In 2017, Li et al. performed a study on 63 patients with severe sepsis and they used the ratio of Il-10 to lymphocyte count as a predictor of 28-day mortality and severity. This ratio showed moderate sensitivity and specificity (around 70%) in their study (16). Expired group had significantly lower lymphocyte count than the survivors.

Wyllie in 2004 and Chien et al. in 2012 suggested that both lymphocyte and neutrophil counts should be considered in adults with suspected bacteremia (13, 14). They showed a quantitative association between lymphopenia and the risk of bacteremia.

Based on the results of the present and the mentioned study, it seems that lymphopenia could be considered as a predictor of 28-day mortality and bad outcome of sepsis patients presenting to emergency department.

## Conclusion:

Based on the findings, lymphopenia was independently associated with higher 28-day mortality and lymphopenic patients were older than the control group and had a significantly higher need for ICU admission, higher probability of 28-day septic shock and readmission due to sepsis, and higher SOFA score. 
